# Effects of relaxation therapy on maternal psychological state, infant growth and gut microbiome: protocol for a randomised controlled trial investigating mother-infant signalling during lactation following late preterm and early term delivery

**DOI:** 10.1186/s13006-019-0246-5

**Published:** 2019-12-16

**Authors:** Jinyue Yu, Jonathan Wells, Zhuang Wei, Mary Fewtrell

**Affiliations:** 1 Institute of Child Health, University College London, London, UK; 2 Child Care Centre, Beijing Children’s Hospital, Beijing, China

**Keywords:** Breastfeeding, Maternal stress, Infant growth, Infant behaviour, Relaxation therapy, Gut microbiome

## Abstract

**Background:**

Breastfeeding is of great importance for infant health both short and long term, especially for those born preterm. Apart from the socio-economic and cultural factors which may influence a mother’s decision on breastfeeding, lactation performance is also influenced by maternal physiological and psychological condition, as well as infant behavioural factors. The aim of this project is to investigate physiological, psychological and anthropological aspects of ‘signalling’ between mother and infant during lactation in a stressful situation, following late preterm delivery, using an experimental approach.

**Method:**

A single blind parallel randomised controlled trial will be conducted in Chinese primiparous mothers who deliver a infant (34 0/7–37 6/7) weeks and plan to exclusively breastfeed. Mothers will be recruited from four local community clinics attached to Beijing Children Hospital. Two home visits will be arranged at one week and eight weeks postpartum. Participants will be randomly assigned to either intervention arm or control (no intervention) before the first home visit. Mothers from the intervention group will be asked to listen to an audio recording with relaxation meditation daily during breastfeeding. Maternal stress and anxiety will be measured at one week and eight week postpartum using Chinese version of Cohen’s Perceived Stress Scale (PSS) and Beck Anxiety Inventory (BAI). Infant weight and length gain (as SD scores) from one to eight week will be measured using anthropometry. Milk volume will be measured using 48-h test-weighing method. Breast milk samples and mother and infant’s stool samples will be collected to measure macronutrient and microbiome content. Anthropometric measurements (weight, length and head circumference) will be performed during all home visits.

**Discussion:**

Primary outcomes of this study will be the effect of the intervention on maternal psychological state, and infant growth. Other outcomes will include the effect of the intervention on milk production, infant behaviours, and the microbiome composition in breastmilk and maternal and infant’s gut. Results of this study will provide greater understanding about maternal-infant factors which influence the success of breastfeeding, and which may then be useful targets for future interventions.

**Trial registration:**

ClinicalTrials.gov identifier: NCT03674632. Registered 14 September 2018.

## Background

Early infancy is a critical period of development and has an important impact on long term health and development. Just as the milk of thousands of other mammalian species has been shaped by natural selection for their offspring, human breast milk has been shaped by selection to maximise the reproductive fitness of human mothers, by promoting the survival and fitness of all her offspring [1, 2]. Increasing evidence shows the effects of human breast milk on optimizing infant growth and development, as well as protecting against infection and developing the immune system [1–4]. However, despite a number of health programmes designed to promote breastfeeding, it is widely recognised that the exclusive breastfeeding (EBF) rates in many countries are disappointingly low and resistant to change [2]. Overall, less than half of infants globally are exclusively breastfed for six months, with a global rate of 39% reported by UNICEF (2012) [5]. In rural areas of China, the EBF rate for infants in the first six months was only 28.7% [6].

Apart from the socio-economic and cultural factors which may influence a mother’s decision on breastfeeding, lactation performance is also influenced by maternal physiological and psychological condition, as well as infant behavioural factors [7, 8]. Breastfeeding is a dynamic process that involves complex physiological signalling and behavioural ‘negotiation’ between the mother and the infant. The baby can ‘signal’ its needs to the mother by its behaviour, such as crying, and the mother can respond by allowing or restricting breast access to alter the production of milk. These processes may shape infant behaviour and feeding, and hence may influence infant growth [7].

While a number of attempts to improve breastfeeding rates focus on providing additional support, many aspects of the breastfeeding process remain poorly understood [2, 9]. Moreover, most studies in this area are observational, which makes it difficult to define causal effects of potential ‘signals’. However, a previous study, the MOM study [10], investigated mother-infant signalling during breastfeeding in 58 Malaysian mothers who were breastfeeding their healthy term infant in a randomised controlled trial; maternal psychological state was manipulated in the intervention group using a relaxation meditation tape. The intervention was effective in reducing maternal stress during breastfeeding, favourably affecting breast milk composition and positively influencing infant sleeping behaviour and growth [10]. Compared to the sample population of this study, there might be greater maternal stress for mothers who have a late preterm infant (LPI) or early term infant (ETI), and the expected benefits of the intervention might be greater. Whilst most LPIs and ETIs who are otherwise healthy are able to establish breastfeeding, the situation is likely to be less straightforward than term infant (>38 week) [11], introducing greater stress. This new study will therefore focus on mothers with LPIs and ETIs, using an experimental approach and a larger sample size to further investigate the causal relationships between maternal psychological state and infant outcomes, and to explore signalling between mother and infant during breastfeeding.

In this study, named the Breastfeed a Better Youngster (BABY) Study, we hypothesise that the use of a relaxation therapy by breastfeeding mothers of late preterm or early term infants starting one week postpartum will result in reduced maternal stress and anxiety, greater infant growth, increased total amount of human milk oligosaccharides in breast milk, increased breast milk volume and energy.

**Fig. 1:**
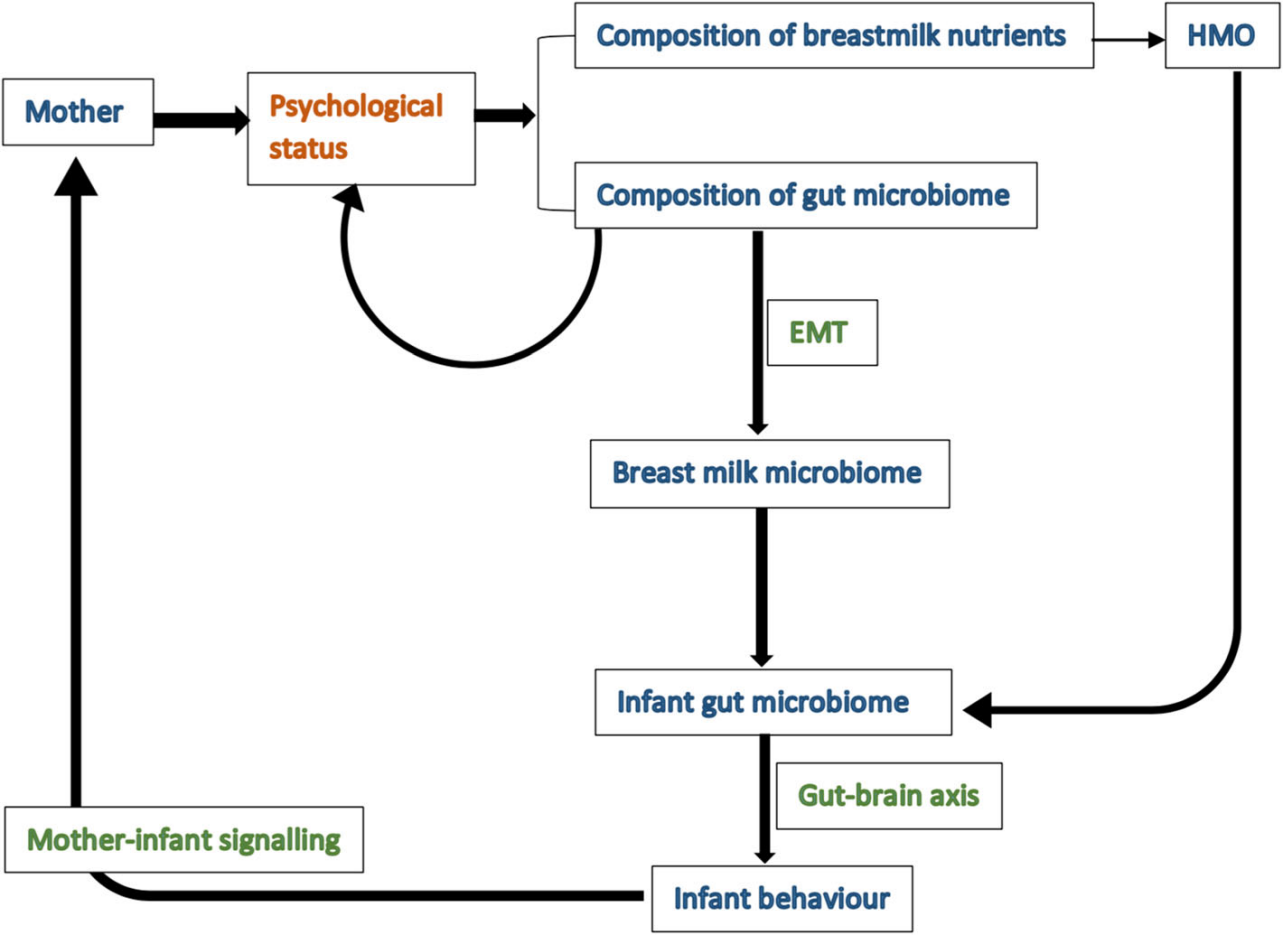
Hypothesis of the mother-infant signalling. Figure Legend – *EMT* Entero-Mammary Trafficking, *HMO* human milk oligosaccharides

In addition, based on recent research, we will investigate the role of the microbiome in mother-infant signalling. The composition of milk microbiota may reflect the mother’s gut microbiome, and in turn influence the microbiome in the infant’s gut [12–14]. Increasing evidence suggests that there is a bidirectional communication between the central nervous system (CNS) and the gastrointestinal tract, which means while the gut microflora can alter the host’s behaviour, the host’s psychological status may also have an influence on modulating the structure of the gut microbiota community [13, 15]. Animal studies using 16S RNA sequencing techniques examined the gut microbiota in male mice/rats and demonstrated that stress can alter the brain-gut axis function and also modifies the relative diversity of the gut microbiota [16, 17]. Studies also supported a role of the gut microbiota in the regulation of stress, emotion, and cognition by using germ-free animals exposed to antibiotic drugs, probiotic bacteria or pathogenic bacterial infections [18–20]. These findings are consistent with the hypothesis that maternal stress during breastfeeding could influence the mother’s gut microbiota, thereby altering the milk microflora, and consequently having an impact on the infant gut microbiota, which could result in changes of infant behaviour (Fig. 1). Thus, the microbiome may play a role in signalling between mother and infant.

## Methods

### Study design

A single-blinded randomised controlled trial will be conducted in Chinese mothers who deliver an infant (34 0/7–37 6/7 weeks of gestation) and plan to exclusively breastfeed. Mothers will be recruited while they are in the maternity hospital. A baseline assessment will be conducted during the one week postpartum home visit, with another study visit at eight weeks after delivery. After obtaining written informed consent, participants will be randomly assigned to either the intervention arm or control conditions (standard management). Participants will be told that the aim of the study is to investigate factors that may make breastfeeding easier for mothers with an LPI or ETI, so they can breastfeed for longer. They will not be told about the randomisation until the end of the study, as this knowledge would most likely lead to mothers in the control group using some form of relaxation therapy. Background characteristics of mothers and their early feeding experiences will be recorded.

### Hypotheses and outcome measures

#### Primary hypotheses

The use of a relaxation therapy by breastfeeding mothers of LPI and ETI that will be given from one week to eight week postpartum, will result in:

1. reduced maternal stress and anxiety
2. greater infant growth, specifically increased weight and length gain

Primary outcomes and measures

1. maternal stress and anxiety at one week and eight week postpartum, measured by Chinese version of Cohen’s Perceived Stress Scale (PSS) and Beck Anxiety Inventory (BAI) respectively.
2. infant weight and length gain (as SD scores) from 1- to 8-week measured using anthropometry.

#### Secondary hypotheses

1. The use of a relaxation therapy by breastfeeding mothers of LPI and ETI from one week to eight week postpartum will result in increased breast milk volume, energy carbohydrate and total amount of HMO in breast milk.
2. The use of a relaxation therapy by breastfeeding mothers of LPI and ETI from one week postpartum will have a positive effect on maternal attitudes toward breastfeeding and infant eating behaviour.
3. The composition of microbiome in vaginally delivered infant’s gut and their mother’s milk is associated, and this will be altered by the intervention, with effects on infant appetite, behaviour and temperament.

Secondary outcomes and measures

1. energy content of breast milk at eight weeks, calculated by milk intake assessed using test weighing methods [21].
2. composition of macronutrients, specifically the lactose and HMO in breast milk
3. composition of breast milk microbiota, maternal and infant gut microbiota, measured by the 16S rRNA based amplicon sequencing technique
4. infant appetite at eight week, three month, and six month, assessed using the Baby Eating Behaviour Questionnaire (BEBQ) [22].
5. maternal feeding attitudes at eight week, three month, and six month, measured using the IOWA Infant Feeding Attitudes Scale (IIFAS) [23].
6. infant behaviour, measured by three day infant behaviour diary at eight week.

### Study population and recruitment

The study population will be recruited from four local community clinics located in different districts of Beijing. The target sample population is Chinese primiparous mothers with a singleton pregnancy who are breastfeeding their LPI or ETI. Eligible mothers (Table 1) will be approached 2–3 days after delivery when they stay at the hospital and breastfeeding is successfully established. The information sheet will be provided to mothers who are interested. A research assistant will explain the outline of the study but will not mention the randomisation. Mothers will be asked to read through the information sheet carefully and discuss with their family.

**Table 1:** Eligibility criteria for mother and infant

Inclusion criteria	
Chinese primiparous mothers	
Singleton pregnancy	
Free from any disease that will influence breastfeeding	
Non-smoker	

**Fig. 2:**
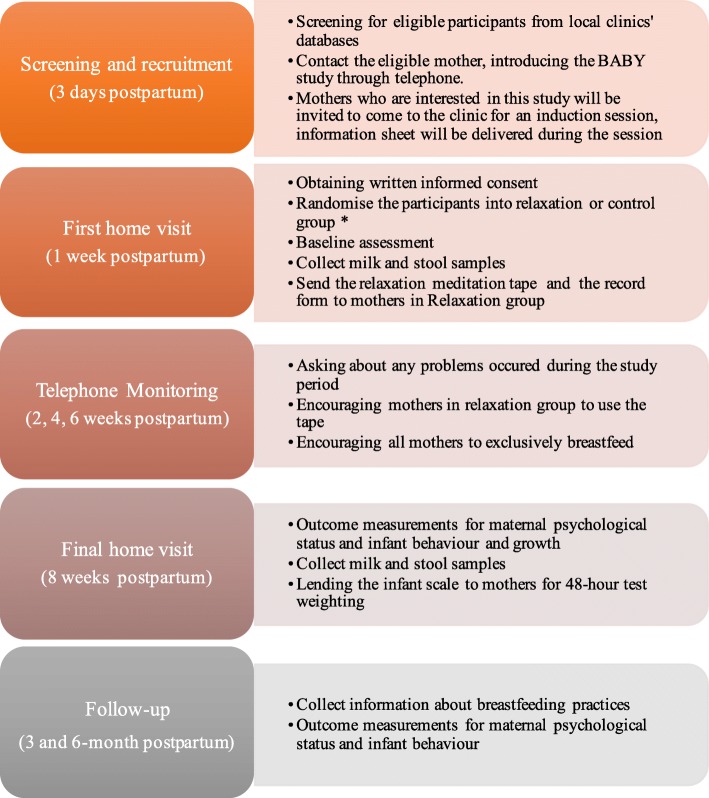
Overview of the study process. Figure Legend – * Participants will not be informed about the randomisation process until the end of the study

After discharge from the hospital, the parents will be contacted by the local clinic to see if they are interested in taking part and the first home visit for baseline assessment will be scheduled at the one-week postpartum follow-up. Eligible participants will be asked to sign the consent form. All questionnaires and the information sheets are in Chinese. An overview of this study is provided in Fig. 2.

### Sample size

The number of mother-infant pairs required was calculated using the conventional formula [24] for two sample t-test:

- *N* = 16*(SD^2^/D^2^) (N = number per group, SD = standard deviation, D = Difference between group).

The SD and D were obtained from the results of the MOM study [10], which assessed the effects of relaxation meditation tape on reducing maternal stress assessed by PSS between intervention and control groups (D = 3.13, SD = 5.00). A sample of 82 mother-infant pairs (41 per randomised group) would allow the detection of a 3.13 points difference in perceived stress between groups at 80% power with a significance level of 0.05. Considering the microbiome analysis in the present study will only be conducted in vaginal delivered mothers, which account for approximately 40% of the total sample, a larger sample is required. However, the effect size in the present study may be larger than the MOM study, as mothers with preterm infants are likely to be more stressed than mothers with healthy term infants, which were examined in the MOM study. Thus, a total of 120 infants will be recruited.

### Randomisation and blinding

The baseline assessment will be conducted during the one week postpartum home visit. After recording the demographic characteristics of mothers and their early feeding experience, participants will be randomly assigned to either intervention arm or control conditions (standard management) by randomisation envelope. A member of the research team who will have no contact with participants will produce the randomisation schedule using a computer random number generator, and prepare the assignments in sealed opaque envelopes. Randomisation will be stratified by gestational age (34–35 weeks, 36–37 weeks), delivery mode (vaginal, caesarean), and by the study centre (four community clinics). Participants will not know about the randomisation until the end of the study; they will be aware that the aim of the study is to investigate factors that may make breastfeeding easier for mothers with an LPI or ETI, so they can breast-feed for longer. The researchers will be blinded during the data analysis process; however, they cannot be blinded during data collection since additional materials (diary for recording the use of relaxation tape) will be collected during the data collection period.

### Data collection

**Fig. 3:**
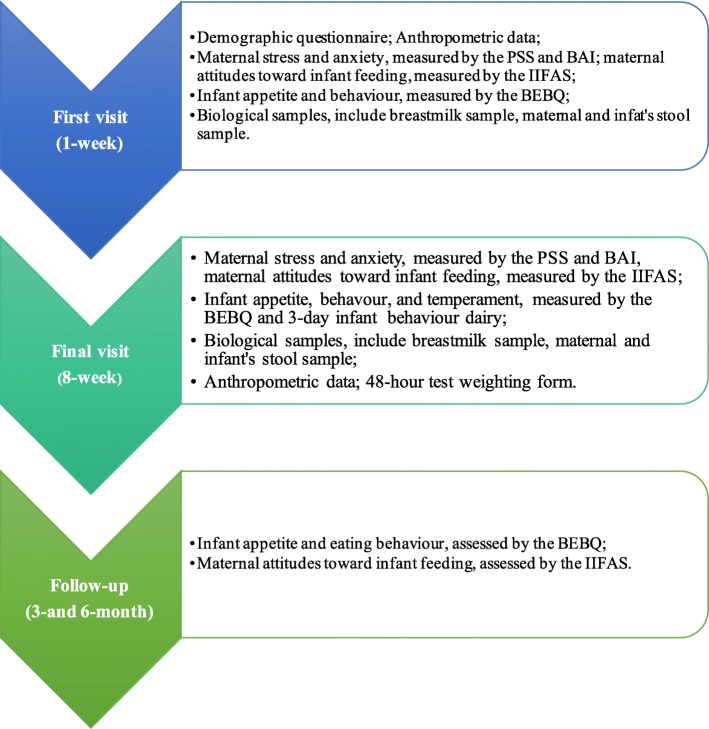
Home visit during the data collection. Figure Legend – *BAI* Beck Anxiety Inventory, *BEBQ* Baby Eating Behaviour Questionnaire, *IIFAS* Iowa Infant Feeding Attitude Scale, *PSS* Perceived Stress Scale

Measures of maternal stress, anxiety, infant behaviour and appetite will be recorded at each home visit; participants can choose to fill the questionnaires on paper or online in their own time after the visit. A breast milk sample will be collected pre-feed and infant anthropometry will be assessed by a trained nurse pre-feed at each home visit. Feed duration will be noted by the trained nurse. Details about the data collection in each visit are shown in Fig. 3.

### Intervention tool: the relaxation therapy recording

The tape used in this study is based on a meditation CD designed for breastfeeding mother [25]. The recording was transcribed and translated into Chinese language by a certified yoga therapist. A brief version of the recording was tested and compared with other four relaxation techniques in a pilot study [26] and was demonstrated to be the most effective approach for breastfeeding mothers to relax. Mothers in the intervention group will be given the tape and will be asked to listen to the recording as frequently as possible while breastfeeding or expressing milk, preferably at least once a day. Mothers will be asked to record their use of the tape in a diary book.

### Questionnaires

Questionnaires that will be used in this study are outlined in Table 2. All questionnaires are available in Chinese. The Chinese version of PSS, BAI, BEBQ, and IIFAS have been used among postnatal women in previous studies, which have presented reasonable validity and reliability.

**Table 2:** Questionnaires for the BABY study

Questionnaire	Time point for the use of questionnaires			
	First visit 1 week	Final visit 8 week	3 month follow-up	6 month follow-up
Demographic questionnaire	✓			
Perceived stress scale	✓	✓		
Beck Anxiety Inventory	✓	✓		
Baby Eating Behaviour Questionnaire	✓	✓	✓	✓
Iowa Infant Feeding Attitudes Scale	✓	✓	✓	✓
3 day Infant Behaviour Diary	✓	✓		

#### Cohen’s perceived stress scale (PSS)

PSS is a psychological self-rating scale for measuring the perception of stress on a scale of five, from 0 (never) to 4 (very often). The original 14-item English version of PSS was developed by Cohen and his colleagues. As a global measure of perceived stress, it appears to be reliable and validated for the measurement of stress in chronic conditions. The present study will use the 10-item Chinese version of PSS, which has been validated for the Chinese population (Cronbach’s α = 0.85–0.86) [27, 28].

#### Beck anxiety inventory (BAI)

BAI is a 21-question multiple-choice self-reported inventory that consist of 21-items that is used to measure the severity of anxiety from different aspect on a scale of four, from 0 (not at all) to 3 (severe). Research demonstrated that the BAI have high internal consistency and reliability (Cronbach’s α = 0.94). The present study will use the translated Chinese version of BAI, which has been validated for the Chinese population (Cronbach’s α = 0.74–0.87) [29, 30].

#### Baby eating behaviour questionnaire (BEBQ)

BEBQ is derived from an existing psychometric measure validated for older ages, the Children’s Eating Behaviour Questionnaire, supplemented by a review of the literature on milk-feeding behaviours. It has been used in a large birth cohort study in the UK (*n* = 4804), and appears to be reliable with Cronbach’s alpha values ranging from 0.73 to 0.81 [22]. BEBQ can be used to measure infant appetite and eating behaviour during the period of exclusive milk feeding, which makes it well-suited for the neonates. It consists of 18 items designed to measure four traits: “enjoyment of food” (4 items), “food responsiveness” (5 items), “slowness in eating” (4 items), and satiety responsiveness” (5 items). The mothers in this study will be asked to rate all items based on a scale from 1 (never) to 5 (always).

#### The Iowa infant feeding attitude scale (IIFAS)

The questionnaire is designed to measure maternal attitudes toward infant feeding methods. It consists of 17 questions and the mother will be asked to give their opinion based on a scale from 1 (strongly disagree) to 5 (strongly agree). This questionnaire has been used extensively and has been tested for reliability with Cronbach’s alpha ranging from 0.62 to 0.74 [31, 32].

#### 3-day infant behaviour diary

Infant crying behaviour will be recorded at one and eight weeks home visit using a validated three day diary. The diary consists of a time scale for 72 h, which is divided into 15 min segments, and has five categories of behaviour: Sleeping, Awake and content, Fussy, Crying and Feeding [33, 34].

### Other assessments

#### Anthropological measurements on mothers and infants

Mothers and infants will be weighed at the one week and eight week home visit. The weight will be measured to the nearest 0.1 kg using an electronic scale. Anthropometric measures on the infants also include recumbent length and head circumference. Each measure will be repeated three times and the mean value will be used. All measurements will be done following the detailed instructions provided by the World Health Organization instructions on child assessment.

#### Test-weighing

For the measurement of milk intake, researchers will lend a digital electronic infant weight scale to mother for 48 h and mother will be asked to weigh her infant right before and right after a feeding, and the duration between the two weight measurements will be recorded to adjust for insensible water loss. Care will be taken to ensure that the infant is weighed with the same clothing before and after the feed. The difference in weight, adjusted for insensible water loss during the feeding period, is the amount of breastmilk the infant consumed during the feeding.

### Sample management and analysis

#### Breastmilk samples

Milk samples (foremilk) will be collected at first and final home visits. Samples will be collected separately for the analyses of milk composition and microbiota. Some milk will also be stored for measurement of milk hormones, as part of other research ongoing in our research group. The collection will be conducted by either manual expression or using a hand pump (Philips Avent, Netherlands). All samples will be stored at -80°C until analysis.

#### Stool samples

Mother and infant stool samples will be collected at first and final home visits by trained nurse and kept in a white capped opaque specimen jar. The specimen jar will be provided by the company with stabilizer in it to keep the microbiome stable. Each jar will be labelled with the baby’ name, date and time of the sample was collected. All samples will be stored at -80°C until analysis.

#### Analysis of milk composition

The macronutrient content will be measured using the Mid-infrared milk analyser (MIRIS, Sweden) at the research centre of Beijing Children’s Hospital, which will present the content of fat, protein, and total carbohydrate of the breastmilk.

#### Analysis of milk energy content

The energy content in breast milk will be estimated from the milk volume measurement using published energy values from the MOM study and the measured energy content in the sample for macronutrients analyses using the MIRIS.

#### Analysis of microbiota

The composition of microbiota in breast milk and faecal samples will be examined using the 16S rRNA based amplicon sequencing technique. The 16S rRNA based amplicon sequencing enables identification of the entire microbial community within a sample up to the species level, which can identify detailed information on the bacterial composition.

### Statistical analysis plan

All questionnaires and anthropometric data will be analysed using IBM SPSS (version 24). Intention-to-treat analysis will be performed for comparing primary outcomes (perceived stress and anxiety, infant weight and length gain from baseline to the endpoint) between intervention and control groups at the eight week postpartum using two sample t-test. Paired t-test will be used to compare baseline to post-intervention changes of each primary outcome. The association between the effects of the intervention and the frequency of use will be examined by two-tailed Pearson correlation. Two-sample t-test will be used to compare the changes in milk volume, energy and macronutrients level between intervention and control group. Regression analysis will be used to adjust for confounding factors.

Furthermore, associations between infant temperament/behaviour and the composition of microbiota in mother’s breastmilk, mother and infants’ gut will be examined using univariate ANOVA. Multiple regression analysis and MANOVA will be used to detect differences among significant predictor variables identified by ANOVA.

## Discussion

One of the strengths of our study is that it has been designed to minimize disruption for the participants since the data collection points have been timed to coincide where possible with routine clinic visits and home visits. According to the health policy in China, the maternity leave is 98 days. During this time, the majority of mothers will practice a traditional postpartum confinement at home and keep routine contact with the community clinic where they are registered. In Beijing, most community clinics will schedule a routine home visit at one week postpartum for all registered mothers. Hence, the baseline assessment of this study is scheduled at one week postpartum when the trained clinic nurses conduct their routine home visit. Thus our baseline assessment and data collection can be conducted during their routine home visit to reduce imposing an extra interruption and additional burden on the mother. Moreover, since there is a routine contact, the nurse can provide the scale and the eight week questionnaires during the contact one week prior to the eight week data collection. Mothers can fill in the questionnaires using their leisure times and return the scale and questionnaires when they take their infant to the clinic for the second month vaccination; the nurse can also conduct the eight week sample collection and infant anthropometry measurement during the time when mothers bring their infants to the clinic for vaccination. These routine contacts with local clinics should help to reduce the potential attrition rate.

In the MOM study [10], the milk intake was measured by the stable isotope method (deuterium dilution). However, the local investigators thought that this would discourage Chinese mothers from participating. Hence, we elected to use the conventional 48-h test-weighing method for the measurement of milk intake, by calculating the difference between the infant’s weight immediately before and after each breastfeeding during a 48-h period. Data will be adjusted for any extra water consumed, water derived from oxidation of milk macronutrients, and water stored in fat and protein (calculated from body water and weight change and published values for protein and water contents of lean body mass) during analysis. A previous study compared the accuracy of test-weighing and the stable isotope method, and showed no significant difference between the two methods by paired t-test [35]. However, both methods might have potential sources of error. The calculation of water output depends on the assumption that the mean loss of water subject to fractionation applies to all infants. A mean value based on recent data in Chinese infants will be used to reduce the bias. Moreover, based on previous research, a 100% error in this figure will only result in 1% error in water output [35].

To control for the influence of antibiotics on maternal gut and milk microflora, the assessment of microbiota will be conducted in mothers who were vaginally delivered their infants, since the delivery mode has been shown to influence infant’s gut microbiota. We will also record the antibiotic use of all mothers and infants at the baseline and follow-up assessments. While the microbiota can be influenced by maternal diet, this would be expected to be balanced between treatment group and control group.

In summary, this study aims to show causal relationships between maternal psychological state and breastfeeding outcomes, which may mediate infant behaviour and the gut microbiota. To our knowledge, the present study will be the first RCT that investigates the role of HMO and microbiota in mother-infant signalling. Findings of this study may identify modifiable factors which can be used to encourage and support exclusive breastfeeding.

## Data Availability

Not applicable.

## Abbreviations

BABY: Breastfeed a better youngster study
BAI: Beck anxiety inventory
BEBQ: Baby eating behaviour questionnaire
EBF: Exclusive breastfeeding
EMT: Entero-mammary trafficking
ETI: Early term infant
HMO: Human milk oligosaccharides
IIFAS: Iowa infant feeding attitude Scale
LPI: Late preterm infant
MANOVA: Multivariate analysis of variance
MOMS: Mother-offspring milk study
PSS: Perceived stress scale
RCT: Randomised controlled trial
